# Shaping dental contract reform: a clinical and cost-effective analysis of incentive-driven commissioning for improved oral health in primary dental care

**DOI:** 10.1136/bmjopen-2016-013549

**Published:** 2016-09-08

**Authors:** C Hulme, P G Robinson, E C Saloniki, K Vinall-Collier, P D Baxter, G Douglas, B Gibson, J H Godson, D Meads, S H Pavitt

**Affiliations:** 1 Academic Unit of Health Economics, Leeds Institute of Health Sciences, University of Leeds, Leeds, UK; 2 School of Oral and Dental Sciences, University of Bristol, Bristol, UK; 3 School of Dentistry, University of Leeds, Leeds, UK; 4 Division of Epidemiology & Biostatistics, Leeds Institute of Cardiovascular & Metabolic Medicine, University of Leeds, Leeds, UK; 5 Unit of Dental Public Health, School of Clinical Dentistry, University of Sheffield, Sheffield, UK; 6 Director of the Dental Translational and Clinical Research Unit, School of Dentistry, University of Leeds, Leeds, UK

**Keywords:** HEALTH ECONOMICS, PUBLIC HEALTH

## Abstract

**Objective:**

To evaluate the clinical and cost-effectiveness of a new blended dental contract incentivising improved oral health compared with a traditional dental contract based on units of dental activity (UDAs).

**Design:**

Non-randomised controlled study.

**Setting:**

Six UK primary care dental practices, three working under a new blended dental contract; three matched practices under a traditional contract.

**Participants:**

550 new adult patients.

**Interventions:**

A new blended/incentive-driven primary care dentistry contract and service delivery model versus the traditional contract based on UDAs.

**Main outcome measures:**

Primary outcome was as follows: percentage of sites with gingival bleeding on probing. Secondary outcomes were as follows: extracted and filled teeth (%), caries (International Caries Detection and Assessment System (ICDAS)), oral health-related quality of life (Oral Health Impact Profile-14 (OHIP-14)). Incremental cost-effective ratios used OHIP-14 and quality adjusted life years (QALYs) derived from the EQ-5D-3L.

**Results:**

At 24 months, 291/550 (53%) patients returned for final assessment; those lost to follow-up attended 6.46 appointments on average (SD 4.80). The primary outcome favoured patients in the blended contract group. Extractions and fillings were more frequent in this group. Blended contracts were financially attractive for the dental provider but carried a higher cost for the service commissioner. Differences in generic health-related quality of life were negligible. Positive changes over time in oral health-related quality of life in both groups were statistically significant.

**Conclusions:**

This is the first UK study to assess the clinical and cost-effectiveness of a blended contract in primary care dentistry. Although the primary outcome favoured the blended contract, the results are limited because 47% patients did not attend at 24 months. This is consistent with 39% of adults not being regular attenders and 27% only visiting their dentist when they have a problem. Promotion of appropriate attendance, especially among those with high need, necessitates being factored into recruitment strategies of future studies.

**Strengths and limitations of this study**

- This is the first published research which explores health outcomes from a preventive focused dental service delivery model.
- The research shows that patients in the practices with the blended contracts significantly reduced gingivitis as measured by bleeding on probing when compared with a traditional service model. This is the first study to confirm that a contract that incentivises prevention can demonstrate a health improvement outcome.
- Neither the practices nor the participants in the study were randomised which is likely to lead to a degree of bias, given that all the practices were self-selected.
- The reduction in bleeding on probing was 10%; however, the effect is likely to be larger as the study had a large number of patients lost to follow-up. The high loss to follow-up, while a limitation of the study, exemplifies the challenges of running a dental practice in order to improve patients' oral health; from this perspective, the data have greater external validity than a highly controlled study with artificially high rates of reattendance.
- The study was also the first to examine the cost-effectiveness of a preventive focused contract. The cost to the commissioners of the service was higher than the traditional model, but more research is needed to assess whether this up-front investment is recouped into the long term and longer than the 24 months study follow-up period.

## Introduction

In the UK, there is an increasing trend to the use of financial incentives. This is evidenced in National Health Service (NHS) primary care, including dentistry.^1^ Within dentistry commissioning, over the past decade has seen dental contracts moving away from payment for units of dental activity (UDAs) towards incentive-driven blended contracts that link with key performance indicators such as access, quality and improved health outcome.^2^ This has included (as part of the Department of Health Dental Contract Reform Programme) a series of national NHS dental contract evaluations exploring how focus can shift from treatment and repair to prevention and oral health by introducing of a new clinical pathway and new remuneration models.^2^ Preliminary findings from the pilots have focused on patient and practitioners views of the new clinical pathway, reporting them to be strongly supportive.^3^ More recent findings focus on adaptation to the new system but also report positive indications about clinical benefits in terms of a reduction of risk and health improvement (the latter measured a basic periodontal examination). However, there are few data from outside the pilot sites that allow comparison of the relative effects of the different contracts.^4^

Evidence of the effectiveness of contracting and incentives in health provision is still emerging with mixed results.^1^ ^5^ ^6^ There are questions over their long-term effects on health outcomes, especially since important activities that lack a target may be underemphasised.^7^ ^8^ Within dentistry, evidence suggests that changes to incentive structures can have a substantial impact on dentists' behaviour, particularly their treatment prescribing patterns^9^ shifting towards treatments with high rewards relative to costs, as opposed to selecting on the basis of clinical factors alone. But, again, the evidence is mixed^10–12^.

A blended/incentive-driven commissioning model for dental services was introduced in West Yorkshire, designed to incentivise quality and oral health improvement in addition to treatment volume. In total, 60% of the contract value was apportioned to delivery of a set number of UDAs. The remaining 40% was dependent on the delivery of quality (systems, processes, infrastructure 20% and oral health improvement 20%). The blended/incentive-driven contracts were configured to encourage the following: the delivery of evidence-based preventive interventions in line with identified needs for a defined population; greater access to dentistry and care being provided by the most appropriate team member. Accordingly, all the practices working under the new contract employed dental hygienists or therapists to provide treatments within their remit. The new contract was built around a care pathway approach in which all patients had an Oral Health Assessment on joining the practice and at each subsequent recall. Four sets of information (age group, medical history, social history (self-care, habits/diet) and clinical assessment) were used to inform a traffic-light (red, amber, green (RAG)) system indicating whether patients are at high (red), medium (amber) or low (green) risk of oral disease. The care pathway was tailored to the risk category of the patient and evidence-based preventive advice and interventions^13^ and on completion and risk-based recall interval.^14^

The aim of this study was to evaluate the clinical and cost-effectiveness of the blended/incentive-driven contract.

## Methods

Using a non-randomised controlled design, the study included three dental practices working under the blended contract and three working under the UDA (traditional) contract. Practices working under the traditional contract were included in the study as comparators and matched with practices working under the new blended contracts by deprivation index, age profile, size of practice and ethnicity. All practices were based in West Yorkshire, UK.^15^ ^16^ RAG was not undertaken in traditional practices and two of them employed a therapist or hygienist.

### Clinical effectiveness

To assess clinical effectiveness for patients receiving care from practices working under the new blended contracts versus those receiving care under the traditional contracts, the primary outcome was the percentage of sites per patient with bleeding on probing (BoP). Secondary outcomes included the following: caries (assessed using the International Caries Detection and Assessment System (ICDAS)),^17^ percentage of extracted and filled teeth and oral health related quality of life (Oral Health Impact Profile-14 (OHIP-14) total score).^18^ Sample size was powered using BoP. We estimated the SD in percentage sites with BoP across a UK cohort to be 27.5%, assuming a within-patient correlation at baseline to follow percentage sites BoP of 0.5 and a common variance in practices. We assumed a clinically meaningful mean difference in percentage sites BoP baseline to follow-up in practices working under blended contracts of 10%, versus a mean difference in percentage sites BoP baseline to follow-up in practices under traditional contracts of 0%. We fixed a type I error rate of 0.05 and a power of 0.8. A design effect was included to account for clustering of patients within both groups of practices, assuming an intracluster correlation of 0.2. A two-sided two independent samples t-test identified a total of 550 patients to be recruited (allowing for a 10% loss to follow-up).

At the patient level, inclusion criteria were that patients must be aged 16 years and over, willing to be followed up for 24 months and give informed consent, a new patient to the dental practice and able to complete the patient complete questionnaires. Postcode, age and ethnicity of all patients included within the sample were recorded and profiled during the analysis.

The practices opened their lists to recruit new patients and all new patients attending the practice for the first time were invited to participate. Before their scheduled appointment, each was sent a letter of invitation and information sheet. Each gave informed consent to participate in the INCENTIVE study prior to any data collection. Patients completed the EQ-5D-3L^19^ and OHIP-14^20^ at their first visit and at the follow-up visit 24 months later. The dentist undertook the clinical assessment of teeth and gingivae using ICDAS and BoP at both visits. Family/social history was taken at the first visit only. Patients were contacted by the dental practice 6–8 weeks before their 24 months follow-up date to arrange an appointment. Contact assumed a variety of mediums including telephone, short message service (SMS) and letter in order to maximise follow-up. Non-responding patients were contacted at least three times to encourage attendance for the final follow-up appointment. Patient contributors worked as integral members of the research team from conception of the research idea to shape our research questions and aid delivery, project management and final data interpretation through to reporting.

An analysis of covariance approach was used to model the primary and secondary outcome measures with follow-up measurement as the outcome and baseline measurement as a covariate.

### Cost effectiveness

We undertook within study cost-effective analyses for patients receiving care from practices working under the new blended contracts versus those receiving care under the traditional contracts, presenting the results as incremental cost-effective ratios (ICERs) from (i) the commissioners' and (ii) service providers' perspective.

UK NHS primary care dentistry is typically commissioned from NHS providers or corporate for profit or not-for-profit bodies. In order to calculate the per person cost to the commissioners (contractual payments), the financial value of the contracts for all six practices in the relevant years was divided by the total number of patients attending at least one appointment in each year to provide a per person mean cost to the commissioner using data provided by NHS England.

The cost to the provider was made up of the cost of the provision less the contractual payment received. Within the study information was collected on the number and duration of appointments, the type of treatment and the dental professional carrying out the appointment/treatment. This information was provided by the practices from the appointment history of each patient. Material costs such as those for X-ray films or filling materials are shown in table 1. The salaries of the different staff involved in the treatment were obtained from national sources such as the Pay Circular and the NHS Agenda for Change and overhead costs were calculated replicating a previously used method^21^ (see table 1). Contractual payments were made up of the total value UDAs claimed for participants included in the analysis subtracted from the costs.

**Table 1:** Salaries and overheads, cost of materials and laboratory costs

Salary	Per hour	Source			
Dentist	£32.89	Pay circular (M&D) 1/2011			
Therapist	£16.59	NHS Agenda for Change pay scales 2011/2012			
Hygienist	£13.56				
Oral health educator	£11.17				
**Overheads**	**% of income**	**Dentist**	**Therapist**	**Hygienist**	**Oral health educator**
Wages and NI (per hour)	17.42%	£32.89	£16.59	£13.56	£11.17
Overheads (per hour)	12.77%	£24.11	£12.16	£9.94	£8.19
**Material**	**Cost**	**Source**			
X-rays Optimum film	£0.33	Kent Express Dental Supplies. 2014. URL: http://www.kentexpress.co.uk/gb-en/kent/ default.aspx?did=kent			
X-rays Periapical film	£0.28				
Amalgam filling 1 spill	£0.90				
Amalgam filling Dycal (base 13 g and catalyst 11 g)	£21.20				
**Laboratory**	**Cost**	**Source**			
Denture Full upper or lower only with standard teeth (×2 for F/F)	£167.05	MGill. 2011 Price List. 2011. URL: http://mgill.co.uk/Price_list/index.html			
Crown	£116.00				
Bridge	£131.00				

All material costs include VAT and 15% off the Kent Catalogue price. The cost of chemicals used to develop the film is excluded but is minimal.

M&D, Medical and Dental; NHS, National Health Service; NI, National Insurance; VAT, value-added tax.

The cost-effective analyses used OHIP-14 and EQ-5D-3L completed at the first and 24 months follow-up visits. The EQ-5D-3L was used to derive quality adjusted life years (QALYs)^19^ ^22^ but as has been suggested, is insensitive in oral health conditions,^21^ and thus, a second analysis was undertaken using OHIP-14.^18^ ^20^ The analyses uses the incremental cost per unit change in OHIP-14 score and the cost per QALY. ICERs are presented. To account for uncertainty around mean incremental costs and effectiveness, we conducted sensitivity analyses and non-parametric bootstrapping; sensitivity analyses were further carried out to account for uncertainty in the cost values. For the commissioner's perspective, we performed one-way sensitivity analyses by assuming either no change or a 3% increase in the total contract value per (financial) year or a 0%, 10% increase or 10% decrease in the number of patients treated per year. For the analysis from the service providers' perspective, costs were altered by ±20%. While these values are essentially arbitrary, it was considered likely to represent any uncertainty in the cost values.

In all cost-effective analyses, costs and salaries are adjusted for inflation and a price year of 2012 used. A discounting rate of 3.5% was used for costs and outcomes.^23^ For the effectiveness and cost-effective analyses, missing data for the OHIP-14 were imputed using median imputation if 2 or less OHIP-14 item scores were missing.^24^ Participants who had more than 2 components of the OHIP-14 missing or missing EQ-5D-3L at baseline and follow-up were excluded from the cost-effective analyses.

## Results

Recruitment started on 1 June 2012 (the first patient entered the study on 14 June 2012) and finished on 31 January 2013. A total of 550 participants were recruited at baseline, of whom, 291 returned 24 months later for a follow-up. There was little difference in age, gender or ethnicity between those who were included in these analyses and those lost to follow-up, those who were lost were generally younger, more likely to be male and had worse baseline oral health, as measured by BoP (higher mean), although this was more variable (higher SD) (table 2). Those lost to follow-up attended 6.46 (SD 4.80) appointments on average.

**Table 2:** Demographic and baseline outcome measures by follow-up status

	New blended contract practices			Traditional contract practices		
Practice	Practice 3	Practice 2	Practice 1	Practice 6	Practice 5	Practice 4
N	26 vs 14	82 vs 79	20 vs 56	16 vs 21	56 vs 35	70 vs 54
Age in years (SD)	47.11 vs 48.50 (17.58 vs 18.12)	41.18 vs 39.38 (13.51 vs 12.81)	35.25 vs 34.43 (14.27 vs 11.33)	42.50 vs 39.48 (16.85 vs 15.70)	39.05 vs 37.57 (14.47 vs 13.33)	44.13 vs 36.93 (16.34 vs 12.85)
Gender: Male/female	12/14 vs 7/7 (46.15/53.84 vs 50.00/50.00)??	37/38* vs 40/37* (45.12/46.34 vs 50.63/46.83)	7/13 vs 29/27 (35.00/65.00 vs 51.78/48.21)	7/9 vs 8/13 (43.75/56.25 vs 38.10/61.90)	30/26 vs 19/16 (53.57/46.43 vs 54.29/45.71)	30/37* vs 31/18* (42.86/52.86 vs 57.41/33.33)
Ethnicity: White/other	24/2 vs 13/1 (92.31/7.69 vs 92.86/7.14)	49/28* vs 56/23 (59.76/34.15 vs 70.89/29.11)	15/5 vs 34/22 (75.00/25.00 vs 60.71/39.29)	15/1 vs 20/1 (93.75/6.25 vs 95.24/4.76)	40/16 vs 19/16 (71.43/28.57 vs 54.29/45.71)	37/9* vs 22/8* (52.86/12.86 vs 40.74/14.81
% of sites BoP	26.04 vs 32.40 (20.17 vs 18.98)	25.09 vs 34.39 (27.42 vs 48.52)	6.19 vs 7.67 (5.79 vs 8.18)	22.39 vs 32.07 (12.21 vs 21.10)	27.23 vs 38.28 (23.48 vs 36.49)	30.66 vs 40.56 (38.56 vs 42.38)
% extracted and filled teeth	32.63 vs 33.19 (25.56 vs 16.80)	39.39 vs 44.13 (31.96 vs 31.34)	32.36 vs 34.87 (23.74 vs 29.48)	47.41 vs 56.01 (31.08 vs 30.64)	33.97 vs 51.26 (33.11 vs 37.11)	44.96 vs 39.67 (34.16 vs 31.80)
OHIP-14 total score	2.50 vs 5.79 (3.86 vs 5.77)	12.08 vs 15.30 (11.11 vs 13.67)	12.89 vs 11.47 (14.64 vs 9.33)	13.19 vs 10.38 (14.01 vs 13.49)	10.43 vs 18.69 (11.75 vs 13.32)	7.20 vs 10.30 (8.74 vs 10.23)
Risk assessment: Red/amber/green	8/14/4 vs 7/5/2 (30.77/53.85/15.38 vs 50.00/35.71/14.29)	64/13/5 vs 66/11/2 (78.05/15.85/6.10 vs 83.54/13.92/2.53)	15/5/0 vs 44/11/0* (75.00/25.00/0.00 vs 80.00/20.00/0.00)			

Completed follow-up versus loss to follow-up, mean (SD) for continuous variables, n (%) for categorical variables.

*Counts do not add to total due to missing data.

BoP, bleeding on probing; OHIP-14, Oral Health Impact Profile-14.

### Clinical effectiveness

Of the 550 recruited, 529 had BoP recorded at baseline but only 270 at 24 months. Following data cleaning, 188 were included in the BoP analysis (n=90 new blended contract, n=98 traditional contract). For BoP, a 95% CI for the effect size was (3.23%, 17.25%) indicating a positive effect for participants under the new blended contracts, but with considerable uncertainty in magnitude.

For caries, 269 had ICDAS recorded at baseline and follow-up, and after data cleaning 187 were analysed (n=92 new blended contract, n=95 traditional contract). There were fewer extractions and fillings at follow-up among patients in practices under the traditional contract by an average of 4.43% (95% CI 1.34% to 7.52%).

Overall practices with traditional contracts had a higher follow-up OHIP-14 score (n=176) by 3.05 (p<0.01), indicating worse oral health related-quality of life. For the oral health assessment (practices under the new blended contracts only), for those who attended baseline and follow-up (n=111), there was an improvement with 68.47% red at baseline and 44.14% red at follow-up.

### Cost effectiveness

A total of 210 patients were included in the cost-effective analyses (n=108 in blended contract practice group and n=102 in traditional group). Four patients withdrew before their appointment, five patients did not have appointment history data at baseline and/or follow-up (hence, costs could not be estimated) and 289 patients did not attend for the follow-up assessment. From the remaining 252 patients, 42 patients had more than two components of the OHIP missing or missing EQ-5D-3L at baseline and follow-up. The age, gender or ethnicity were similar in those included in these analyses and those lost to follow-up. Of those included in the blended practice group, the mean age was slightly lower (40.66 vs 43.14), there were slightly fewer men (45.6% vs 51%) and fewer patients who recorded their ethnicity as ‘white’ (72.4% vs 79.1%).

The mean number of UDAs per person was 11.23 (SD 8.08) in the blended practices and 10.74 (SD 8.23) in the traditional practices. However, there were negligible differences in the average number of UDAs per person if patients' were seen by a dentist, 10.70 (8.07) and 10.58 (8.25), respectively. It is difficult to draw conclusions for the therapist and hygienist as some of the practices did not employ dental care professionals. Patients in practices with the blended contracts had more appointments than those in traditional practices, mean number of appointments 8.89 (4.50) and 6.63 (2.93), respectively.

The mean per person cost for the blended contact group was higher for the service commissioner (mean per person cost of £459.77 vs £281.57). However, the blended contracts were financially attractive for the dental provider at the practice level (costs less contractual payments equated to a mean per person cost of £−209.26 vs £−116.21, table 3).

**Table 3:** Mean per patient cost to dental providers

	Blended contract practices	Traditional contract practices
Resource	*Mean (SD)*	*Mean (SD)*
Time of dental professionals	£195.63 (102.92)	£107.02 (55.55)
Materials and laboratory costs	£54.88 (114.47)	£58.34 (111.25)
Total	£250.51 (186.85)	£165.37 (146.28)
Payment to providers	£459.77 (278.42)	£281.57 (218.71)
Total mean healthcare costs	£-209.26 (123.36)	£-116.21 (99.16)

No significant difference in EQ-5D-3L scores were found between groups or over time (table 4). Overall, improvement was observed in OHIP-14 scores between baseline and follow-up for both groups. The mean OHIP-14 scores were 7.11 vs 8.00 for the patients in the blended contract practice and traditional practices, respectively. The ICERs are reported in table 5.

**Table 4:** Mean OHIP-14 and EQ-5D-3L scores by group

		Blended contract practices (N=108)	Traditional contract practices (N=102)
OHIP-14 Time point			
Baseline	Mean (SD)	8.99 (10.30)	9.12 (10.98)
Follow-up (24 months)	Mean (SD)	5.60 (7.58)	7.38 (8.89)
Change	Mean (p value)	3.39 (<0.001)	1.74 (0.051)
EQ-5D-3L Time point			
Baseline	Mean (SD)	0.880 (0.250)	0.896 (0.232)
Follow-up (24 months)	Mean (SD)	0.882 (0.207)	0.897 (0.257)
Change	Mean (p value)	−0.018 (0.235)	0.014 (0.552)

OHIP-14, Oral Health Impact Profile-14.

**Table 5:** Cost-effective results

Commissioner's perspective	Costs (£)	OHIP (points)	
	*Mean (SD)*	*Mean (SD)*	
Blended contract practices	459.77 (278.42)	7.110 (7.673)	
Traditional contract practices	281.57 (218.71)	8.005 (8.699)	
	Incremental cost	Incremental OHIP	ICER (£/OHIP)
Blended contract vs Traditional practices	178.20	−0.895	199.22
**Service providers perspective**	**Costs (£)**	**OHIP (points)**	
	*Mean (SD)*	*Mean (SD)*	
Blended contract practices	−209.26 (123.36)	7.110 (7.673)	
Traditional contract practices	−116.21 (99.16)	8.005 (8.699)	
	Incremental cost	Incremental OHIP	ICER (£/OHIP)
Blended contract vs Traditional practices	−93.05	−0.895	−104.03 BLENDED CONTRACT DOMINATES
**Commissioner's perspective**	**Costs (£)**	**QALY**	
	*Mean (SD)*	*Mean (SD)*	
Blended contract practices	459.77 (278.42)	1.659 (0.451)	
Traditional contract practices	281.57 (218.71)	1.660 (0.342)	
	Incremental cost	Incremental QALY	ICER (£/QALY)
Blended contract vs Traditional practices	178.20	−0.0008	BLENDED CONTRACT DOMINATED
**Service providers’ perspective**	**Costs (£)**	**QALY**	
	*Mean (SD)*	*Mean (SD)*	
Blended contract practices	−209.26 (123.36)	1.659 (0.451)	
Traditional contract practices	−116.21 (99.16)	1.660 (0.342)	
	Incremental cost	Incremental QALY	ICER (£/QALY)
Blended contract vs Traditional practices	−93.05	−0.0008	122,089.48

ICER, incremental cost-effective ratios; OHIP, Oral Health Impact Profile; QALY, quality-adjusted life years.

The sensitivity analyses made little difference to the results with the exception of the bootstrapping from the service providers' perspective which produced an ICER of £9483.68 (under the threshold value). However, it should be noted that the ICER is being driven by the incremental QALY; a small increase in the (already small) incremental QALY will have a big impact.

## Discussion

Participants of the INCENTIVE study were recruited from practices that had purposefully opened their practices to new patient enrolment. The blended contract practices were newly established in areas of high need and where there was little existing service, they therefore had a less established patient base. However, the study was limited to new patients only within mainly deprived communities where routine attendance is low. Within the study the primary outcome favoured patients in the blended contract group. The blended contracts were financially attractive for the dental provider but carried a higher cost for the service commissioner. However, the attrition rate was much higher than anticipated. It appears that many patients chose not to return to their dentist for routine follow-up appointments. Recall visits for oral care assessment at 24 months were poorly attended despite great efforts by the practice staff to invite the patients. Such patients were more likely to be younger males and have poorer oral health. Although the reasons for non-attendance recorded by the dental practices were incomplete, of those recorded, the most frequently cited was no response to contact from the dental practices. In the blended/incentive-driven practices, there was also a substantial number who failed to attend prebooked appointments. Those lost to follow-up had had multiple appointments and treatment including fillings, crowns and bridges (on average 6.46 appointments). One possible explanation for non-attendance at recall may be that the problem for which they had originally visited had been rectified, and in the absence symptoms, they did not wish to visit the dentist for a ‘check-up’. This aligns with data that 27% English adults only go to the dentist when they have a problem.^2^ The proportion in this study is likely to be larger as they were not already regular attenders. Previous studies have seen much lower attrition rates,^25^ but such studies often recruit regular attenders rather than those recruited in this study who were new patients and who may have different drivers for attending the dentist. The low rate of reattendance for regular dental care (thereby implying a pattern of emergency care) provides support for the care pathway approach recommended in the Steele Report, which legitimises irregular dental attendance for those who choose it.^2^ High proportions of patients adopting this pattern will shape the practices in these areas. Such practices will be characterised by the provision of more emergency care and more of the time-consuming initial assessments. This burden needs to be considered in the commissioning of services in these areas.

The attendance rate for the study's 24 month assessment has confounded the conclusions that can be drawn with respect to the relationship between the contract types and clinical outcome. The primary clinical outcome, BoP, favoured the blended/incentive-driven model of service delivery, but the results should be treated with caution given the high attrition and issues of data quality. BoP was selected because it is responsive to change and readily measured. It can be changed by dental teams' behaviours including preventive advice and treatment but is also subject to other changes in patients' behaviours and measurement error. However, it is likely that the influence of patient behaviours and measurement error would be random and would attenuate the apparent relationships between the contract types and the patient outcome. The estimates of the treatment effect may therefore be underestimated in this study. It is possible that measurement bias may have favoured one or other contract type, but this seems unlikely. The utility of BoP as a surrogate for other oral health outcomes warrants further investigation. It should be noted that data quality for BoP and dental caries was an issue with a substantial number of participants excluded from the analysis following data cleaning. These issues included problems with impossible transitions between baseline and follow-up and missing data. Further investigation is required to confirm how to improve the validity of clinical outcome measures in primary dental care research. Dentition charting was highlighted as a problem within the national Dental Contract Pilots, where early findings revealed incomplete or absent charting for many patients.^3^ Perhaps accurate charting could be used as a quality criterion in the blended contracts.

The health economic analyses showed the blended/incentive-driven contract was more costly for the commissioner. Overall oral health-related quality of life (OHQoL), assessed using the OHIP-14, was higher for participants in the blended/incentive-driven practices than it was in practices under the traditional UDA-based contract, but it did not represent a minimal important clinical difference.^26^ The differences within and between groups for the EQ-5D-3L were negligible. This latter result was not entirely unexpected. While the EQ-5D is the National Institute for Health and Care Excellence (NICE)^23^ recommended outcome of choice for economic evaluation, its apparent insensitivity to oral health conditions led to the use of the OHIP-14.^21^ The EQ-5D-3L has been reported to have adequate construct and convergent validity, but appears to be less sensitive than specific measures of OHQoL.^27^ ^28^

The study design reported here enabled a direct comparison between practices offering incentive-driven preventative dentistry and those offering traditional solely activity-driven contracts. These data enhance early findings from the national Dental Contract Pilots following the Steele Report, which have focused on patient and practitioners views of the new clinical pathway, reporting them to be strongly supportive.^3^ Recent findings from the national programme have focused on adaptation to the new system and positive indications about clinical benefits in terms of a reduction of risk and health improvement (measured through the RAG and a basic periodontal examination). This study adds value to the current evidence base of blended/incentive-driven contracts, being the first to systematically evaluate the impact of a dental service provision on oral health outcomes. It compares those operating under the existing volume-based contract and those, driven in part by incentives that have been developed in partnership with the dental practices.

One of the challenges in undertaking the study was its pragmatic study design. Neither the practices nor the participants in the study were randomised. There will inevitably be a degree of bias, given all the practices were self-selected. The three blended contract practices were newly established and had competitively tendered to operate practices under the new contract, and as such may be thought of as early adopters and may well be atypical of dental practices in England. Similarly, while the traditional contract practices were matched to the blended/incentivised contract practices, they chose to participate. One stumbling block for existing traditional practices was that all the participants had to be new patients. For some practices, this was not viable and such those practices declined our invitation to participate. The matched practices and high attrition also meant that two of the three matched practices were unbalanced in terms of participant numbers.^15^ This leads us to question whether it is robust to pool the three pairings in single analyses. Although there is some reassurance that the effect size for the primary outcome (BoP, pooled across practices) is similar to that included in the original power calculation (10.24% observed vs 10% in calculation) and achieves statistical significance (p<0.01), this cannot guarantee the study achieved power of 80% for the primary outcome as originally specified in the study design.

## Conclusions

Overall, the evidence of the effectiveness of use of contracting and incentives in health providers is still emerging with further experimental research needed; specifically, the impact on patient outcomes.^10^ This is the first UK study to assess the clinical and cost-effectiveness of a blended contract in primary care dentistry providing an important perspective by which to inform the dental contract reform. Although the findings overall with respect to the clinical effectiveness of the blended/incentivised contracts are mixed, the results of the primary outcome of gingivitis (BoP) favour the blended/incentivised model, and there is some reassurance that the effect size is similar to that included in the original power calculation. The economic analysis showed the blended/incentive-driven contracts arm of the study to attract a higher cost for the dental commissioner but to be financially attractive for the dental provider at the practice level. The results should be treated with caution given the sample attrition. Recommendations are to factor in higher attrition rates into future primary care dental studies especially those of high social deprivation where regular attendance is not the usual pattern of service access.
